# A review of cutaneous manifestations of HTLV-associated adult T-cell leukemia/lymphoma

**DOI:** 10.3389/fmed.2025.1603102

**Published:** 2025-09-26

**Authors:** Tessa M. LeWitt, Alejandro A. Gru

**Affiliations:** ^1^Department of Dermatology, Columbia University, New York, NY, United States; ^2^Division of Dermatopathology, Columbia University, New York, NY, United States

**Keywords:** ATLL, HTLV-1, HTLV, leukemia, lymphoma

## Abstract

Adult T-cell leukemia/lymphoma (ATLL) is a rare aggressive mature T-cell neoplasm caused by the human T-cell leukemia virus type 1 (HTLV-1). The disease predominantly affects patients from endemic regions of Japan, the Caribbean, Sub-Saharan Africa, and South America. Cutaneous involvement of ATLL is common and may appear before or in the absence of systemic involvement. However, the lesions are non-specific and often mimic more common inflammatory and neoplastic skin diseases, making diagnosis challenging. Moreover, morphology is variable among patients, ranging from macules and papules to tumors and erythroderma. Recent data suggests that the presence of cutaneous lesions and lesion morphology are important for prognostication and for guiding treatment. Here, we provide an overview of the spectrum of cutaneous findings in ATLL, highlighting their diagnostic, prognostic, and therapeutic implications. We emphasize the importance of early recognition and workup of suspicious skin lesions, particularly in endemic regions or high-risk populations. Finally, we describe fundamental concepts and controversies, and highlight current knowledge gaps.

## Introduction

### Pathogenesis

HTLV-1 is an oncogenic retrovirus implicated in several human diseases including ATLL, HTLV-1 associated myelopathy/tropical spastic paraparesis (HAM/TSP), and several inflammatory conditions of the eye and skin. The virus is estimated to infect about 10 to 20 million people worldwide and its prevalence varies by region, with endemic areas identified in parts of Japan, Sub-Saharan Africa, the Caribbean, and South America. HTLV-1 is most commonly transmitted vertically via breastfeeding but can also be transmitted through sexual contact or by exposure to infected blood products (1).

HTLV-1 primarily infects CD4^+^ T-cells through direct cell-to-cell transmission, leading to lifelong infection. While most HTLV-1 carriers remain asymptomatic throughout their lives, an estimated five percent of carriers will ultimately develop ATLL (2). First described by Uchiyama et al. in 1977, ATLL is an aggressive mature T-cell neoplasm found predominantly in HTLV-1 endemic areas (3). Mean age of diagnosis is 62 years without a sex predilection (4).

The pathogenesis of ATLL and other HTLV-1 related diseases is complex, and much is yet to be elucidated. Nonetheless, the pivotal importance of HTLV-1 encoded regulatory proteins such as Tax and HTLV-1 basic leucine zipper factor (HBZ) in ATLL pathogenesis and progression are well established. Tax is a potent transcriptional activator that induces genetic instability, modulates intracellular signaling pathways, and enhances HTLV-1 infected T-cell proliferation and transformation. Tax is highly immunogenic, making it susceptible to killing by cytotoxic T-lymphocytes, and is therefore only expressed in bursts (5, 6). Moreover, Tax expression is disrupted or lost in a majority of ATLL cases, particularly in aggressive subtypes (7).

HBZ functions to promote mitotic activity of HTLV-1 infected cells by several mechanisms including suppression of Tax gene transcription and, in some cases, by causing infected cells to take on a regulatory T-cell-like phenotype. HBZ is significantly less immunogenic than Tax and is therefore able to maintain low-level constitutive activation without eliciting a strong host immune response. Unlike Tax, HBZ transcripts are not lost in ATLL (5, 6).

HTLV-1 proviral load (PVL) refers to the number of host T-cells carrying the integrated HTLV-1 provirus in their genome. PVL is significantly higher in those with ATLL compared to asymptomatic carriers, reflecting the increase in viral load prior to malignant transformation (8, 9). Most studies suggest that PVL is highest in patients with aggressive ATLL subtypes, however PVL alone is not predictive of aggressive disease (8, 10, 11). PVL is particularly relevant with regards to the cutaneous manifestations of ATLL, as patients with clonal integration of HTLV-1 proviral DNA in their skin lesions progress more rapidly and exhibit lower medial survival times compared to their counterparts without proviral integration (12).

### Classification and workup

In 1991, Shimoyama et al. proposed a classification system for ATLL based on clinical features, laboratory findings, and probability of disease progression. Despite some controversy and proposals for modification, the Shimoyama classification remains the most frequently used classification system for ATLL. It recognizes four distinct subtypes: acute, chronic, lymphoma, and smoldering (13). Of these subtypes, the acute and lymphoma subtypes are considered aggressive with median survival of less than 1 year. Despite being classified as “indolent,” chronic type (with favorable features) and smoldering type have median survivals of only 5.4 and 2.9 years, respectively. Additionally, nearly half of patients within “indolent” subtypes ultimately transform into the acute type within less than 2 years (14).

Cutaneous lesions are commonly encountered in all clinical subtypes of ATLL but are most common in chronic and smoldering subtypes (15, 16). Skin lesions may present at any time in the disease course and can occur anywhere on the body (17–19). Clinical presentation is heterogeneous and non-specific, often mimicking common skin diseases (e.g., atopic dermatitis and psoriasis) and cutaneous malignancies [e.g., mycosis fungoides (MF) and Sézary syndrome (SS)] (17, 20, 21). Systemic symptoms (e.g., fever, night sweats), lymphadenopathy, or hepatosplenomegaly accompanying these skin lesions may help point the clinician toward an underlying neoplastic etiology, but bloodwork and histopathologic evaluation are always necessary to establish the diagnosis. Notably, the presence of hypercalcemia in association with a skin lymphoma should always prompt for the evaluation of HTLV-1 serology.

ATLL can present with a variety of symptoms, and while the most common clinical manifestations are systemic, cutaneous involvement is frequently observed (15). Understanding the cutaneous manifestations of ATLL and their histopathologic correlates is essential for clinicians who encounter this condition as they can provide important diagnostic, prognostic, and therapeutic information.

## Cutaneous manifestations of ATLL

The majority of cutaneous findings in patients with ATLL can be categorized as patches, papules, plaques, nodules, tumors, purpuric lesions, or erythroderma, though rates of each presentation vary widely in individual reports (18, 19, 22, 23). Rare presentations such as lichen planus-like lesions, vesiculobullous lesions, hypopigmented patches, and ichthyosis have also been described (16, 17, 20, 24, 25). Multiple morphologies often coexist in the same patient (19, 26). In contrast to MF, lesions are not characteristically localized to the photo-protected “bathing trunk” areas. While most studies identify the trunk and extremities as the most frequently involved sites, a cohort of 126 Jamaican patients found localized lesions more commonly on acral surfaces (16). Lesions can also be seen in usual locations such as the lip and nasal vestibule (19, 20).

### Macules, patches, papules, and plaques

**FIGURE 1:**
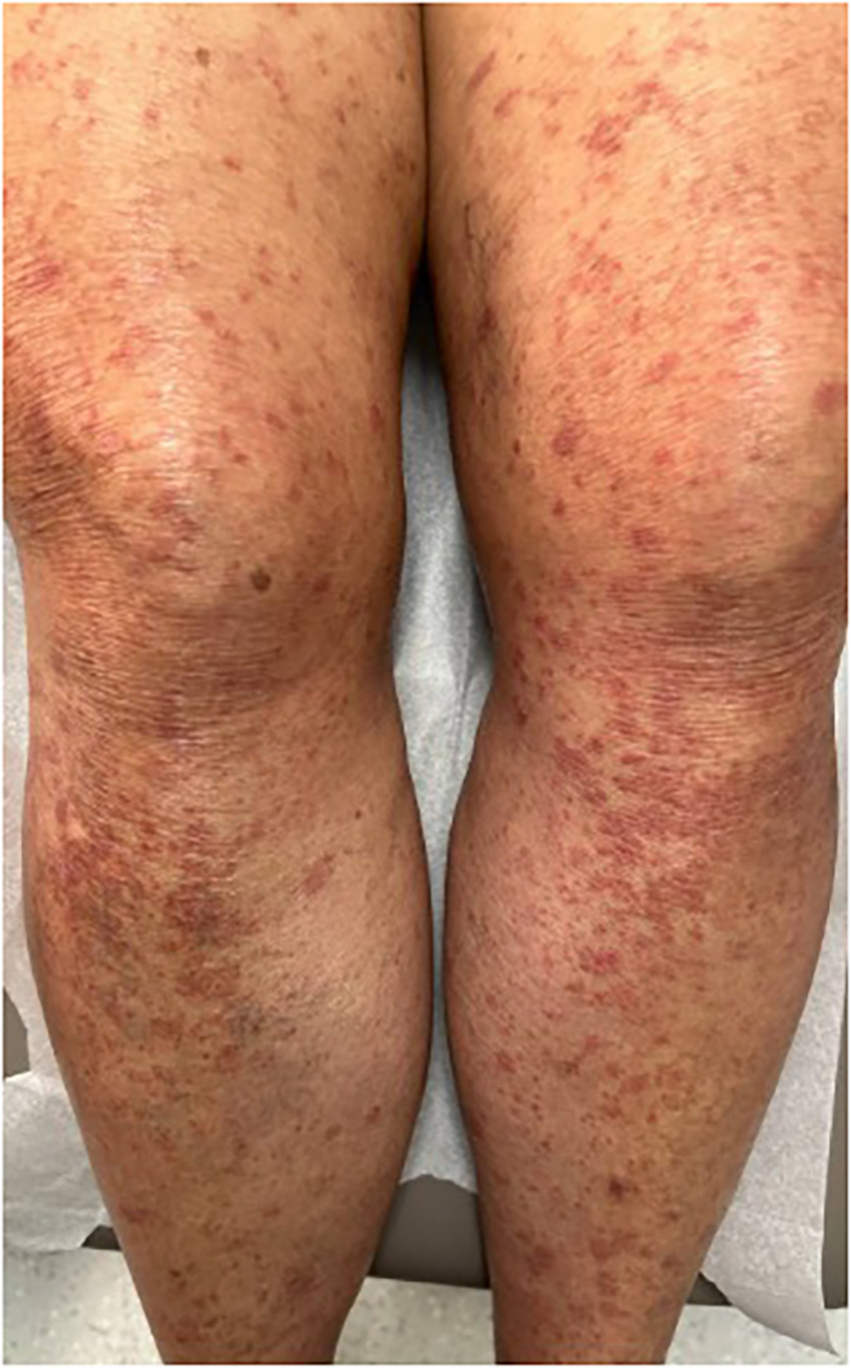
Red to brown macules coalescing into patches on the lower legs.

**FIGURE 2:**
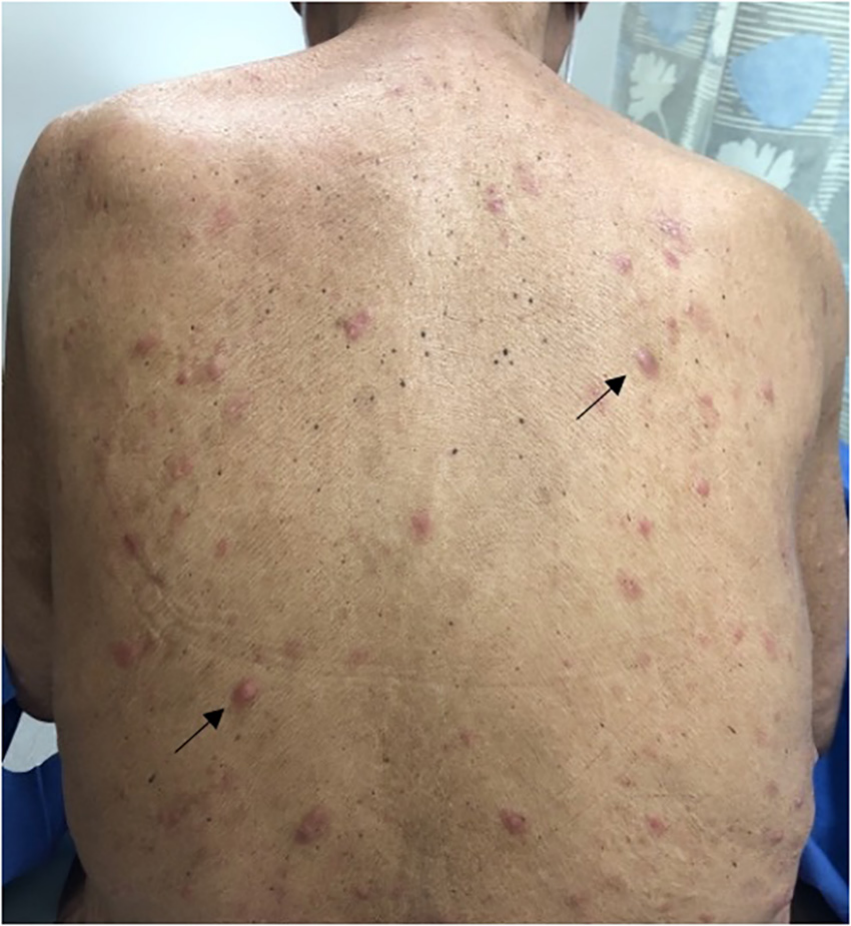
Pink indurated papules scattered throughout the back (black arrows).

**FIGURE 3:**
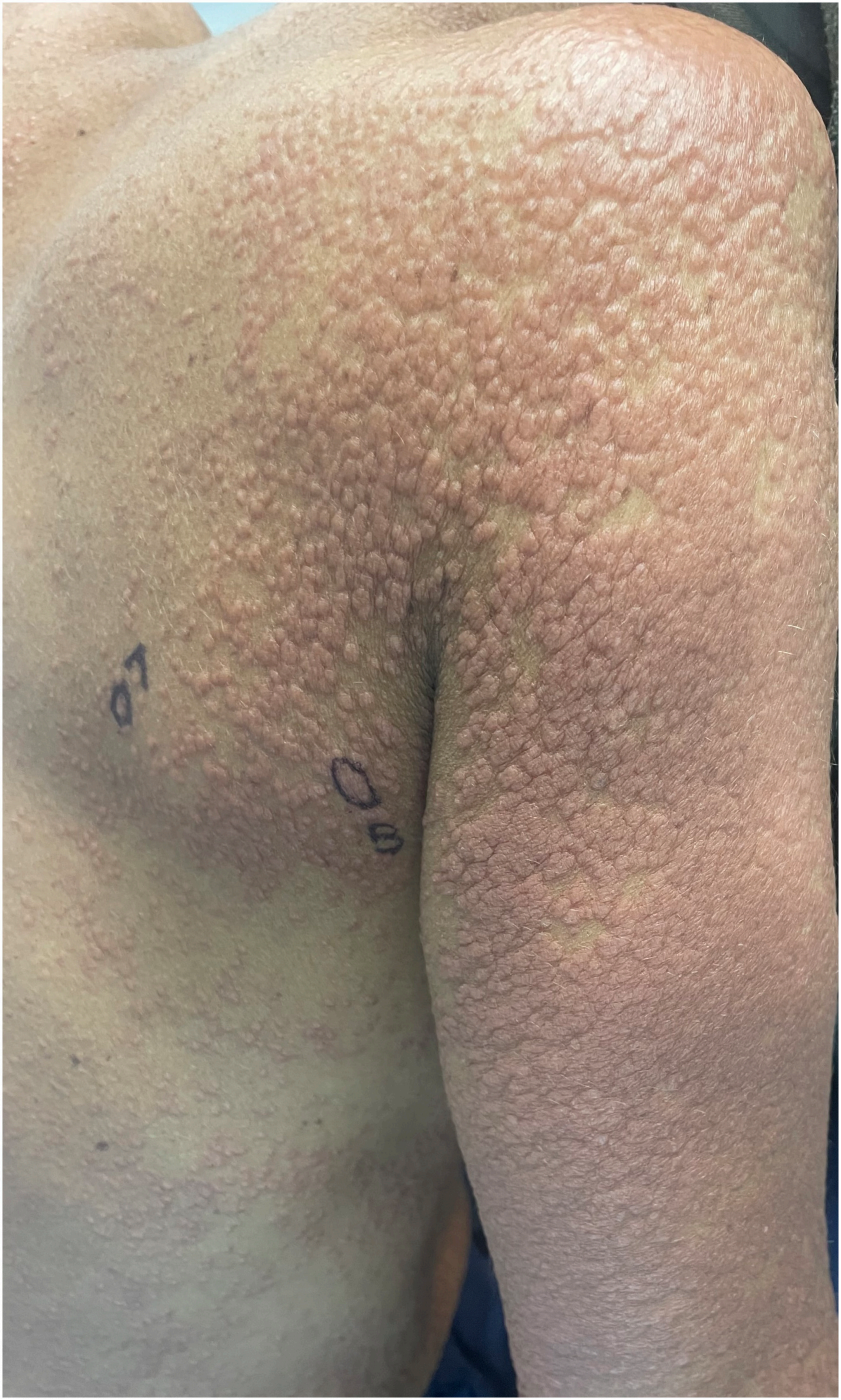
Diffuse pink to brown papules on the right posterior shoulder and arm coalescing into plaques.

**FIGURE 4:**
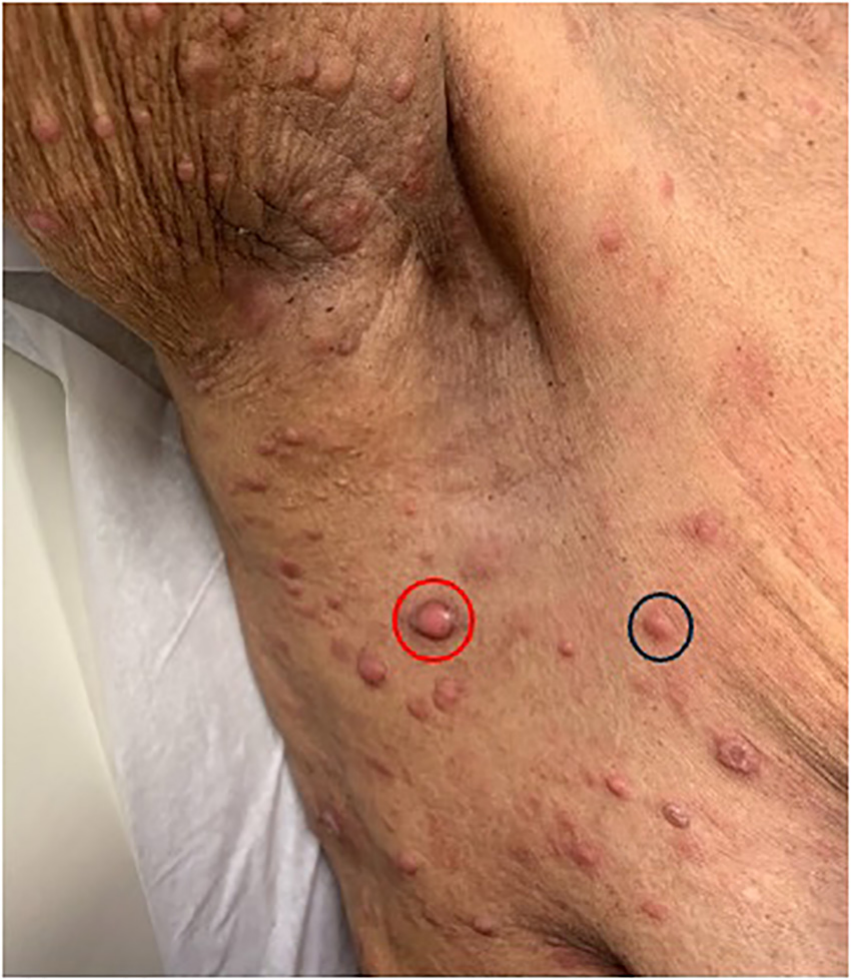
Pink papules in the axillary region (black circle) with one larger central nodulotumoral lesion (red circle).

Studies suggest that macules, patches, papules, and plaques are the among the most frequently encountered skin manifestations of ATLL worldwide and may, in some cases, be associated with a relatively favorable prognosis (Figures 1–4) (15, 18, 26, 27). Macules and patches are defined as flat, non-palpable lesions, with macules measuring less than or equal to 1 centimeter (cm) and patches measuring larger than 1 cm. Papules and plaques are raised, palpable lesions similarly distinguished by their size (papules ≤ 1cm, plaques > 1cm). These lesions can present anywhere on the body and can be singular, multiple, or diffuse (18, 28).

A Japanese cohort of 124 ATLL patients with skin lesions, demonstrates the prevalence of these lesion types in all ATLL subtypes, reporting that macules, papules, and plaques accounted for 66.6% of skin manifestations in the acute subtype, 47.6% in the chronic subtype, 26.9% in the lymphoma subtype, and 53.6% in the smoldering subtype (17). Similarly, in a Japanese cohort of 99 patients with chronic or smoldering type ATLL treated by dermatologists, patches, multi-papular eruptions, and plaques were observed in 25, 25, and 9% of patients, respectively (15). It is important to note that in this cohort, skin involvement with acute and lymphoma subtypes was rare (<3%) compared to chronic and smoldering subtypes (21.9 and 48.4%, respectively) (15). Thus, one must understand prognostication of skin eruption type within the context of ATLL subtype (15, 18). For example, in a Brazilian cohort of patients with ATLL and skin lesions, papules and plaques were the most common lesion types observed in those with the acute subtype. Although no association was found between the type of skin lesion and prognosis, patients with acute-type ATLL, as expected, had a poorer prognosis (27).

### Erythroderma

Erythroderma, defined by diffuse erythema affecting greater than 80% of the body surface area (BSA) (18), is another important but rare cutaneous presentation that has been observed in all ATLL subtypes (28). Erythroderma is a life-threatening manifestation of several dermatologic diseases (e.g., psoriasis, atopic dermatitis) and is often accompanied by non-specific systemic findings such as fever, lymphadenopathy, and generalized pruritus. The prevalence of erythroderma in ATLL is overall low compared to other skin eruption types, making up less than 6% of ATLL cases with skin involvement (15, 17–20). Not surprisingly, outcomes for erythrodermic patients are poor. In a study of 119 ATLL patients with skin eruptions, Sawada et al. found that the median survival time for erythrodermic patients was the lowest of all eruption types (∼3 months) and was associated with poorest prognosis (18). Importantly, each of the 5 erythrodermic patients in this cohort had acute type ATLL.

### Nodules and tumors

Nodulotumoral lesions, which contain an infiltrate in the dermis or subcutis, present clinically as firm lesions that are larger and more indurated than papules or plaques (Figure 3). Nodulotumoral lesions are found in up to 38% of patients with ATLL and are often indicators of severe disease. For example, in a cohort of 52 ATLL patients with cutaneous involvement, tumors were only seen in aggressive ATLL subtypes (28). The prognostic and therapeutic indications of these lesions are an area of especial clinical interest and debate (15, 18). In 1992, Johno et al. proposed a subtype of “cutaneous type ATLL” that is not associated with either lymph node or leukemic involvement (29). This group further subdivided their patients into two phenotypes: “tumoral” and “erythematopapular,” noting that the tumoral group had a worse prognosis. In 2007, Bittencourt et al. advocated for inclusion of Primary Cutaneous Tumoral (PCT) ATLL into the Shimoyama classification based on characterization of patients with cutaneous nodulotumoral lesions (but without lymphadenopathy, lymphocytosis, hypercalcemia, or internal organ involvement) that had a more aggressive course than patients with smoldering type ATLL without skin lesions (28, 30). Though PCT ATLL patients are currently classified as smoldering type according to the current classification, the 2019 International Revised ATLL Consensus recognized PCT ALL and stated that watchful waiting is inappropriate in cases of PCT ATLL due to its aggressive nature and poor prognosis (31, 32).

Several other groups have corroborated the presence of this distinct aggressive phenotypic subgroup, citing that those with smoldering type ATLL and cutaneous nodules or tumors had a more rapidly progressive course and lower median survival rates than their counterparts without skin lesions (17, 18, 30, 31, 33). In a Japanese cohort of 119 patients with smoldering type ATLL and cutaneous lesions, survival was statistically significantly worse in patients with nodulotumoral disease compared to those with only patches or plaques (15). However, this group found no significant difference in survival between patients with and without skin lesions and therefore concluded that the type of skin lesion (rather than presence of skin lesions alone) may predict prognosis in patients with smoldering type.

Data regarding HTLV-1 PVL provides further evidence to support cutaneous type ATLL as distinct from smoldering type. For example, Yonekura et al. found that HTLV-1 PVL is significantly lower in cutaneous type ATLL than in smoldering type ATLL (34). In another Japanese cohort of 46 patients with ATLL and cutaneous lesions positive for proviral DNA, 9 patients met criteria for cutaneous type ATLL. These nine patients showed poorer prognosis than those with smoldering type but significantly better prognosis than those with other ATLL subtypes (12).

The relationship between cutaneous lesions and worse ATLL outcomes may not only be true for patients with smoldering type. For example, in a study that included patients with all ATLL types, mean survival time was 17.3 months for patients with nodulotumoral and multi-papular lesions compared to 114.9 months for patients with plaques. This group ultimately determined that skin eruption is an independent prognostic factor for ATLL when considering all subtypes together (18).

## Prognosis, staging, and treatment

### Prognosis and staging

One group attempted to stratify patients based on their skin eruptions by applying the TNMB classification for MF/SS to their patients with ATLL. Of the 91 patients who could be classified using this system, 16% were T1 (patch/plaque, <10% BSA), 17.7% were T2 (patch/plaque, >10% BSA), 38.7% were T3 (nodulotumoral), and 4.2% were T4 (erythrodermic) (18, 35). They observed a direct relationship between higher T (tumor) stage and frequency of aggressive types (acute and lymphoma) and an indirect relationship between higher T stage and frequency of smoldering type. Furthermore, as T stage increased, overall survival decreased. They also noted that when compared to patients without skin lesions and patients with stage T1, patients with stages T2-T4 had worse overall survival; there was no significant difference in overall survival in patients with stage T1 and those without skin lesions. One substantial limitation to the utilization of the MF/SS staging criteria to patients with ATLL and skin involvement is the ambiguous staging for patients with multi-papular, purpuric (non-blanching lesions caused by extravasation of red blood cells into the skin), ichthyotic (dry, scaling), or other rare lesion types not recognized by the T classification system for MF/SS.

Median overall survival of patients with cutaneous involvement does not seem to depend on whether patients have skin-first disease (i.e., skin lesions as first presentation of ATLL) or skin second-disease (i.e., skin lesions that develop after ATLL diagnosis) when stratified by ATLL subtype (20). Numerous lesion types have been described in both skin-first and skin-second disease, though Marchetti et al. reported that 6 of their 8 patients with skin-second disease had tumors or nodules at consultation (19, 20). In their cohort, nearly one-third of patients had skin-first disease and all patients with chronic or smoldering type ATLL had skin-first disease. Patients with skin-first disease had significantly longer median duration of symptoms (11.9 months) compared to those with skin-second disease (1.8 months) plus those without skin lesions (1.9 months) (20).

### Treatment

The stratification of ATLL patients with cutaneous lesions is particularly relevant with regards to treatment, as patients with aggressive subtypes (i.e., acute and lymphoma) are candidates for alternate treatment algorithms.

For patients with asymptomatic smoldering disease and no skin lesions, current consensus guidelines recommend active monitoring. In symptomatic smoldering disease (i.e., with skin lesions) and in patients with favorable chronic disease, active monitoring remains an option; however, treatment with zidovudine and interferon-α (AZT/IFN) with or without arsenic trioxide is also recommended when available. If AZT/IFN is not accessible and tumors are present, systemic chemotherapy followed by allogeneic hematopoietic stem cell transplantation (allo-HSCT) should be considered.

For non-tumor skin lesions, skin directed therapy can be utilized in conjunction with active monitoring (32). Skin directed therapy for cutaneous lesions largely depends on lesion type, presence of symptoms, and extent of BSA involvement. Treatment options include topical steroids, narrow-band ultraviolet B therapy, psoralen photochemotherapy, electron beam therapy, radiation therapy, and surgical resection (15).

Due to its aggressive course, acute ATLL should be treated with AZT/IFN when possible, or intensive chemotherapy otherwise, with early allo-HSCT for eligible patients. Lymphoma-type ATLL also warrants intensive chemotherapy, with low-dose AZT/IFN as maintenance when available, and early allo-HSCT for suitable candidates (32). For relapsed or refractory disease, treatment options include single-agent therapies or alternative combination chemotherapy regimens (e.g., etoposide, high dose cytarabine). More recently, clinical trials have explored the use of novel therapies such as the enhancer of zeste homolog (EZH) inhibitor valemetostat, lenalidomide, the histone deacetylase (HDAC) inhibitors tucidinostat, and antibodies and antibody-drug conjugates including mogamulizumab and brentuximab-vedotin (14, 36). These trials are often limited by small sample sizes and variable results; additional research is needed before guideline-supported recommendations can be made. It should be noted that given the aggressive nature of ATLL and relatively low therapeutic success with currently available treatment options, clinical trials should be considered for all patients (14, 32, 33).

## Conclusion

Cutaneous manifestations of ATLL are heterogeneous and non-specific. Nevertheless, they may serve as the earliest presentation of disease and can aid in prognosis and management. The most common skin lesions are patches, papules, plaques, nodules, tumors, and erythroderma but rare presentations and unusual lesion distribution should not steer the clinician away from suspecting ATLL.

A comprehensive approach, including thorough physical exam, histopathological evaluation of skin lesions, and laboratory testing for HTLV-1 remain essential for accurate diagnosis and appropriate treatment. Clinicians should maintain high level of suspicion for ATLL even if the diagnosis remains non-diagnostic after initial biopsy, as many patients are initially misdiagnosed (20). This index of suspicion should be particularly high for patients from HTLV-1 endemic regions such as the Caribbean or Japan. Early identification of ATLL is crucial for timely intervention and improving patient outcomes. Since patients who initially present with cutaneous symptoms often experience a prolonged duration before diagnosis, there is an opportunity to enhance the early identification and outcomes in these cases.

Additional large-scale and prospective studies are needed to more accurately describe the relationships between survival outcomes and presence or subtype of skin lesions, but there is substantial evidence to suggest that a subtype of smoldering type patients with nodules and tumors have worse outcomes than what is typically expected for this “indolent” ATLL subtype. The rarity of ATLL makes the implementation of large-scale studies difficult and necessitates collaboration among institutions.
